# A Stage in the Evolution of Medicine

**Published:** 1895-11-16

**Authors:** 


### The Hospital

An Institutional Journal of

The Medical Sciences and Hospital Administration, with

AN EXTRA NURSING SUPPLEMENT.

Vol. XIX.—No. 477.] [Nov. 16, 1895.

### A Stage in the Evolution of Medicine.

The Rev. Augustus Jessop, D.D., rector of Scarning, Norfolk, who has been called "the genial clerical philosopher of the *Nineteenth Century*"—of the periodical, that is to say, which bears that title—says, "It is becoming plainer and plainer, to some of us that of all the learned professions the medical profession has of late years risen higher than any other in the public estimation, and that the *status* of the medical man is tending to become recognised as entitled to precedence above that of any other class in the community. For myself, I incline to believe that this is inevitable."

Whether the *status* of the doctor is or is not "entitled to precedence above that of any other class in the community" is of little practical importance. What is of importance to ourselves is the fact that our profession has won its way to the ungrudging respect of the community, and what is of an importance common both to ourselves and the community is the means by which this has been accomplished. The doctor is to-day a man of acknowledged authority in his sphere; at the beginning of the century he was not so. How has this all-significant change been effected? The answer is that now, not the best medical men only, but even the average doctor stands before his fellows as a plain embodiment of the mastership of truth and knowledge on the one hand, and perfect simplicity of character and conduct on the other. In other words, the scientific habit of mind has led, not only to assured knowledge, but to decision and authority in character, and manly simplicity in conduct. All these endowments are recognised by the Rev. Dr. Jessop as being of the kingly order, and as tending inevitably to give to their possessors "precedence above that of any other class in the community."

But there is another side to the shield, the more is the pity; and those who recognise responsibility among us, instead of soothing ourselves with the, perhaps, too flattering judgments of our clerical critic, turn our faces to that other side, not without humiliation. It is the fact, as a long correspondence in one of our contemporaries has made plain, that a considerable proportion of men, in the highest as well as in the lowest ranks of our profession, are resolved, by cunning self-advertising and other like devices, to utilise their calling solely as a means of obtaining prominence and wealth. That prominence and wealth are the aims of the majority of commonplace mankind we admit: that they are almost universally considered both legitimate and natural aims we cannot deny. But one thing is certain, namely, that if this order of medical men should prove stronger than the other, and should convert the whole profession into mere seekers of prominence and wealth, the position of weight and authority and of high social precedence, which Dr. Jessop tells us we have so justly gained, will speedily become a thing of the past, a lost nobility whose recovery will be for ever impossible.

Whatever men may say to the contrary this is truth: to aim at knowledge is a high aim; to aim at practical ability to help one's fellow-men is a high aim; to aim at serving one's generation with the one pure motive of doing our patients all the good which the best science of the times enables us to do, is a high aim. In comparison with those aims the resolution to be prominent and rich, and to use our medical knowledge and skill for these sole ends, are as poor and vulgar and shameful as any aims of educated men can well be.

To our thinking the medical profession has reached something of a crisis in its history. Under the influence of a tremendous environment, it is tempted to give up its old ways of strenuously searching for scientific truth, and devoting itself with absolute singleness of purpose to the application of that truth at the bedside. But let it make no mistake. Those ways, which it is now tempted to give up, are the very ways which have made medicine great and famous, and earned for it an ungrudged precedence among all men of culture and worth. It is not to be tolerated that the men of lower aim should overshadow the men of greater worth—men to whom the medical profession owes all it has so tardily acquired of dignity and solid value. Some decisive steps—we cannot at the close of an article indicate what—should be taken by the leading men and institutions of physic to conserve the character of the profession, to make more obvious the true greatness of its methods and aims, and to expose with unsparing logic the sinister designs of those who, for their own private ends, would degrade and ruin one of the noblest callings ever pursued by man.

